# Biomarkers and Echocardiographic Predictors of Myocardial Dysfunction in Patients with Hypertension

**DOI:** 10.1038/srep08916

**Published:** 2015-03-09

**Authors:** Agata Bielecka-Dabrowa, Marta Michalska-Kasiczak, Anna Gluba, Ali Ahmed, Eva Gerdts, Stephan von Haehling, Jacek Rysz, Maciej Banach

**Affiliations:** 1 Department of Hypertension, Chair of Nephrology and Hypertension, Medical University of Lodz, Poland; 2 Department of Nephrology, Hypertension and Family Medicine, Chair of Nephrology and Hypertension, Medical University of Lodz, Poland; 3 University of Alabama at Birmingham, Birmingham, Alabama, USA; 4 Department of Clinical Science, University of Bergen, Norway; 5 Applied Cachexia Research, Department of Cardiology, Campus Virchow-Klinikum, Charité Medical School, Berlin, Germany

## Abstract

The study aimed to identify early echocardiographic and circulating biomarkers of heart failure (HF) in hypertensive patients with normal resting echocardiography. Echocardiography at rest and during exercise, and selected biomarkers were assessed in control group, dyspnea group, and HF group. On exercise dyspnea patients had lower early diastolic (E') and systolic (S') mitral annular velocity (12.8 ± 1.0 *vs* 14.9 ± 3.0 cm/sec and 9.3 ± 2.0 *vs* 10.9 ± 2.0 cm/sec, respectively), and higher E/E' ratio compared to control group (6.7 ± 1.0 *vs* 5.9 ± 1.0) (*p* < 0.05 for all comparisons). The level of N-terminal propeptide of procollagen type III (PIIINP) was significantly higher in dyspnea group than in controls (*p* = 0.01). Control and dyspnea patients had lower levels of cardiotrophin-1, cystatin C, syndecan-4, and N terminal–probrain natriuretic peptide than HF patients (all *p* ≤ 0.01). In multivariate analysis PIIINP (unadjusted odds ratio [OR] = 8.2, 95% confidence interval [Cl] 1.7–40.6; *p* = 0.001; adjusted OR = 8.7; 95%CI: 1.5–48.3; p = 0.001) and E/E' ratio on exercise (unadjusted OR = 1.8, 95%CI: 0.8–4.0; *p* = 0.033; adjusted OR = 2.0; 95%CI: 0.8–4.8; p = 0.012) were the only factors significantly associated with the presence of dyspnea. PIIINP is the first early biomarker for the HF development in patients with HA and normal resting echocardiography. Exertional echocardiography may indicate patients with incipient HF with preserved ejection fraction.

Cardiovascular disease (CVD) is the leading cause of death. Importantly, it remains the foremost cause of preventable death globally^1^. The reason for the enormous burden of hypertension has been reported in numerous studies, showing that hypertensive disease is strongly associated with overall CV risk^1^/Increased blood pressure (BP) contributes indeed to both CV and cerebrovascular endpoints, including heart failure (HF), myocardial infarction (MI), and stroke^1^. As reported in 2013 statistics of the American Heart Association, 33.0% of US adults ≥ 20 years of age have hypertension^1^.

HF prevalence continues to rise in developed countries, particularly in the elderly^2^. HF is a progressive disease with a preclinical phase (stage B HF) characterized by the presence of structural and/or functional cardiac abnormalities that precede the development of the overt disease^3^. Hypertension is a major cause of HF through induction of left ventricular hypertrophy (LVH) and coronary atherosclerosis. LVH is due not only to cardiac myocyte hypertrophy but also to increase in the extracellular matrix and collagen structure^3^,^4^. In hypertensive patients, LVH is often associated with exertional dyspnea. However, hypertensive patients independent of presence of LVH on resting echocardiogram, may have abnormal left ventricular function that may be diagnosed only during exercise and explaining exertional dyspnea^4^,^5^. Hypertension is a main cause of HF with preserved ejection fraction (HFpEF), although this represents a heterogeneous disease^6^. Major pathophysiological factors implicated in HFpEF are impaired relaxation, increased LV stiffness and reduced compliance, atrial dysfunction, chronotropic incompetence, pre- and post-capillary pulmonary hypertension, and vascular stiffening^6^,^7^. Low-grade inflammation and extracellular matrix accumulation and fibrosis are often described, and may significantly contribute to impaired LV filling, a hallmark of the disease^6^,^7^. Although echocardiography is the most useful non-invasive diagnostic method for evaluating systolic and diastolic dysfunction, current state-of-the-art echocardiography has limited value for prognostication in HF^8^. In fact echocardiography at rest may not distinguish between stage B HF patients and HFpEF patients^9^. HF results from a complex interplay between genetic, neurohormonal, inflammatory, and biochemical changes acting on cardiac myocytes and the cardiac interstitium. Thus, the sequence of events that lead to overt changes in the ventricle begins at the cellular level and assessing these phenomena could be of greater value to improve prognostication^9^,^10^. Some important polymorphisms linked to hypertension including calcium/calmodulin-dependent kinase IV and G-protein-coupled receptor kinases open new potential fields of translational investigation to treat patients with hypertension^9^,^10^.

Biomarkers may play an important part in this respect. They may provide important information on the pathogenesis of HF, but may also be a valuable clinical tool in the identification of patients at risk for HF, in the diagnosis of HF, in risk stratification, and in the therapy monitoring^8^,^11^,^12^. There are several biomarkers from different biological pathways reflecting the multi-systemic character of HF, which should be considered based on the analysis of the available literature^8^,^9^,^10^,^11^,^12^: N terminal-probrain natriuretic peptide (NT-proBNP) - biomarker of myocyte stress and gold standard for HF; cardiotrophin-1 (CT-1) - a member of the interleukin-6 (IL-6) family of cytokines; tumour necrosis factor-alpha (TNF-alpha) - a proinflammatory cytokine, IL-1 receptor like protein 1 (IL1R1) - a receptor for proinflammatory cytokine IL-1; cystatin C (CysC) -a low-molecular weight protein from the group of cysteine proteinase inhibitors; transforming growth factor-beta 1 (TGF-beta 1) - a central regulator of cardiac fibrosis; N-terminal propeptide of procollagen type III (PIIINP) - one of the biomarkers of extracellular matrix remodeling; galectin-3 - a protein involved in cell adhesion, cell activation, chemoattraction, cell growth, cell differentiation, fibroblast activation and apoptosis; syndecan-4 - a biomarker of left ventricular remodeling; neutrophil gelatinase-associated lipocalin (lipocalin-2/NGAL) - a glycoprotein involved in cell survival, inflammation and matrix degradation^8^,^9^,^10^,^11^,^12^.

The main aim of the study was to identify echocardiographic markers during exercise and circulating biomarkers characterizing hypertensive patients with exertional dyspnea. This study reviews the hypothesis that circulating biomarkers and exertional echocardiography allow for early identification of hypertensive patients at risk of HF. The study was also designed to try establish the panel of diagnostic tests in patients with hypertension, which will allow for early detection of abnormalities and to clarify the etiology of exertional dyspnea in patients with hypertension and normal resting echocardiography.

## Methods

### Study population

120 patients with stage I or II essential hypertension^13^ were included consecutively in the study between October 2012 to February 2014. The exclusion criteria were as follows: coronary artery disease (CAD) or a history of CAD, unstable hypertension, New York Heart Association (NYHA) class IV HF, percutaneous or surgical revascularization, evidence of pulmonary hypertension on echocardiography, obstructive or restrictive pulmonary disease (groups control and dyspnea, congenital or valvular heart disease, the presence of arrhythmia (including atrial fibrillation [AF]), electrical pacemaker or implantable cardiac defibrillators, hyperthyroidism and hypothyroidism, pregnancy and lactating, hemodynamically significant acquired heart defects, cardiomyopathies (in control and dyspnea groups), glomerular filtration rate (GFR) < 60 ml/min/1.73 m^2^ (in control and dyspnea groups), chronic liver insufficiency, cancer, significant anemia, abuse of alcohol or drugs, chronic inflammatory and other diseases, or lack of informed consent to participate in the study.

Dyspnea was graded by the NYHA functional classification^14^. Angina pectoris was graded by the Canadian Cardiovascular Society (CCS) scale^15^. The venous blood samples were drawn fasting in the morning and the obtained serum was frozen at the temperature of 70°C. Estimated GFR was calculated using the Modification of Diet in Renal Disease (MDRD) formula^16^. Systolic and diastolic arterial pressures were measured using a sphygmomanometer and stethoscope at rest and after exercise.

Approval from the Bioethics Commission of the Medical University of Lodz (No. RNN/749/13/KB) was obtained. All the methods were performed in accordance with approved guidelines. Written informed consent was obtained from all the patients.

### Biomarker tests

The concentrations of NT-proBNP, CT-1, CysC, TNF-alpha, PIIINP, syndecan-4, IL1R1, TGF-beta 1 and lipocalin-2/NGAL were determined using the EMax Endpoint ELISA Microplate Reader analyzer (Molecular Devices; Sunnyvale, California, USA). TNF-alpha was analyzed with the enzyme-linked immunosorbent assay (ELISA) (Diaclone/Gen-Probe, USA), with 2 polyclonal antibodies directed against TNF-alpha. Determination of NT-proBNP and CT-1 was performed with reagents of USCN Life Science Inc./Cloud-Clone Corp (Wuhan, China), using a sandwich ELISA assay according to the manufacturer's protocol. Measurement of CysC was performed using a sandwich enzyme immunoassay (BioVendor, Brno, Czech Republic) developed for the quantitative measurement of this marker in human serum. Analysis of the concentration of PIIINP, syndecan-4 and IL1RL1 was performed with a USCN Life Science Inc./Cloud-Clone Corp (Wuhan, China) kit, using a sandwich ELISA assay according to the manufacturer's protocol. Measurement of TGF-beta 1 was performed using a sandwich enzyme immunoassay (Gen-Probe Diaclone SAS, Besançon, France) designed for the quantitative detection of TGF-beta 1 levels in cell culture supernatants, human serum, plasma or other body fluids. Determination of lipocalin-2/NGAL was conducted using the BioVendor Human Lipocalin-2/NGAL ELISA sandwich enzyme immunoassay. Analysis of the concentration of galectin-3 was performed with a USCN Life Science Inc./Cloud-Clone Corp (Wuhan, China) kit, using a sandwich ELISA assay.

### Echocardiography

All patients were examined following a standardized protocol using an ALOKA Alpha 10 Premier (Tokyo, Japan) with a 3–11 MHz probe.

Quantitative echocardiography was used following current guidelines^17^. Left ventricular volumes and ejection fraction (EF) was determined by biplane Simpson's method. Left ventricular (LV) mass was calculated using the Devereux formula. The early (E) and atrial filling (A) peak velocities, E/A ratio, deceleration time of early filling and isovolumic relaxation time were measured from transmitral flow. Peak systolic (S'), early diastolic (E') and late diastolic (A') mitral annular myocardial velocity of the left ventricle septal and lateral walls was recorded from the apical 4-chamber view with pulsed-wave tissue Doppler and results were averaged. The E/E' was calculated as an index of LV filling pressure. All measurements were made off-line on three consecutive beats and averaged values are reported.

### Exercise testing

Symptom-limited (fatigue, dyspnea or stenocardia) exercise testing was done in groups control and dyspnea on a bicycle ergometer to a HR of less than 100 bpm.

### Statistical analysis

The STATISTICA 10 software package (StatSoft, Poland) was used for analysis. All values presented are the mean ± standard deviation (SD) for continuous variables and number and percentages for categorical variables. To compare two groups, Student's t-test for continuous variables with normal distribution and Mann-Whitney U test for non-normally distributed variables was used. For comparison of three groups analysis of variance (ANOVA) with *post-hoc* Tukey's test for unequal sample sizes or Kruskal-Wallis ANOVA was used as appropriate. Correlations between variables were tested with Spearman's rank correlation coefficient. Bootstrapped logistic regression was used to identify factors significantly associated with the presence of dyspnea. All the echocardiographic images were analysed by the single investigator. The intraobserver variability was performed in 10 randomly selected patients at rest and during exercise and reported as interclass correlation coefficient (ICC) with 95% CI. A value of p < 0.05 was considered significant.

## Results

### General characteristics of patients

There were 150 patients potentially eligible for the study, 120 of patients were examined for eligibility, 21 patients were excluded (2 had CAD, 4 had AF and 15 did not have adequate exercise images for analysis). The remaining 99 hypertensive patients were grouped into 3 groups: 22 asymptomatic hypertensive patients (control group), 27 patients with exertional dyspnea (dyspnea group) and 50 hypertensive patients with overt HF. HF patients were defined using combined criteria: clinical symptoms and LVEF <50%. Patients in control and dyspnea groups had normal resting echocardiography. Because patients with hypertension are more likely to develop coronary artery disease^18^, dyspnea group included only patients with negative results of treadmill exercise testing and who had undergone computed tomography coronary angiography without any identified changes in the epicardial coronary arteries and Agatston score 0.

The patients' characteristics are presented in Table 1. Compared to asymptomatic patients, hypertensive patients with dyspnea more frequently reported stenocardia. Compared to HF patients, hypertensive patients with dyspnea had significantly higher BP, MDRD GFR, and calcium channel blockers and sartans were used more frequently in this group. In successive groups there was an increase in degree of HF according to NYHA class.

### HF biomarkers in hypertensive patients

**Figure 1:**
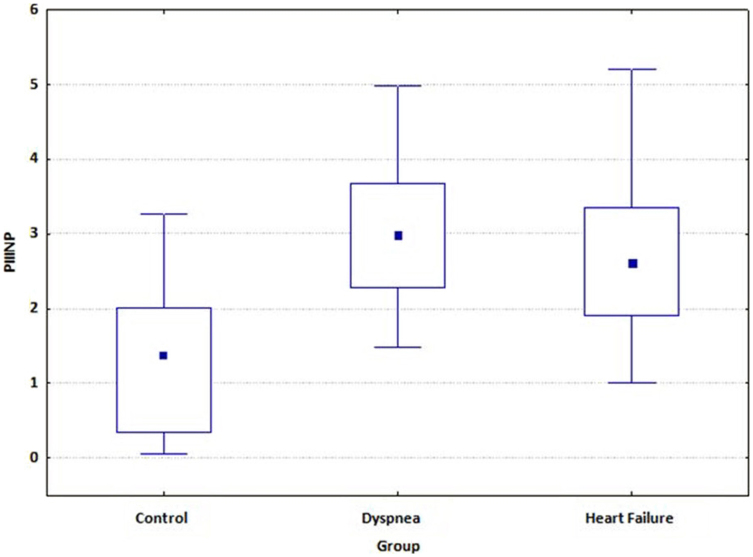
Concentrations of PIIINP in the investigated groups. **ABBREVIATIONS:** PIIINP – collagen III N-terminal propeptide.

Higher concentrations of collagen III N-terminal propeptide were observed in patients with hypertension with dyspnea compared to asymptomatic patients with hypertension (2.9 ± 0.94 *vs* 1.27 ± 1.03; *p* = 0.0001) (Table 1), concentrations of PIIINP did not differ between hypertensive patients with dyspnea and HF patients (Figure 1). Compared to the asymptomatic hypertensive patients, hypertensive patients with dyspnea had similar values of other biomarkers. Compared to HF patients, hypertensive patients with dyspnea had significantly lower values of CT-1, syndecan, NT-proBNP, CysC, TGF beta and NGAL (*p* < 0.0001; *p* = 0.0015; *p* < 0.0001; *p* = 0.0001; *p* < 0.0001 and *p* = 0.036 respectively). The detailed data on biomarkers in investigated groups is presented in Table 1.

### Echocardiographic findings at rest

Compared to the asymptomatic hypertensive patients, hypertensive patients with dyspnea had longer isovolumic relaxation time (*p* = 0.033). Compared to dyspnea group, patients with HF had larger dimensions (*p* < 0.0001), LV volumes (*p* < 0.0001), lower LVEF (*p* < 0.0001), greater wall thickness of the left ventricle (*p* < 0.02), increased LV mass and LV mass index (*p* = 0.001), lower E', S', increased E/E' ratio (p < 0.05) and significantly increased left atrial (LA) diameter (*p* < 0.0001) (Table 2).

### Findings during exercise echocardiography

**Figure 2:**
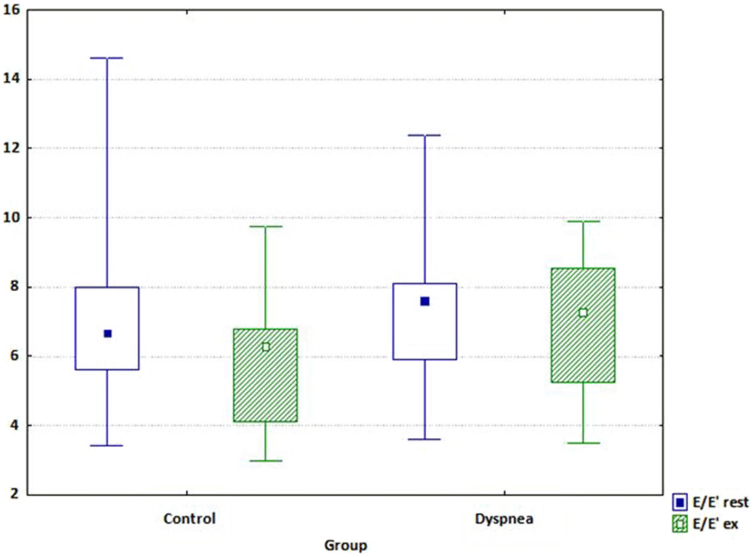
Values of E/E' in both groups (Control and Dyspnea) before and during exercise.

The increase in E′ with exercise was significantly smaller in patients with symptoms compared with control subjects with Δ E' (*p* = 0.004) and the increase of E/E' ratio (*p* = 0.017; *p* = 0.020; and *p* = 0.034, respectively). On exercise systolic and early diastolic velocities of mitral annulus were increased both in control and dyspnea group and the values were significantly higher in control group than in dyspnea group (*p* = 0.02; *p* = 0.01, respectively) (Table 3). There was a significant decrease in the E/E' after exercise only in control group (*p* = 0.02). Figure 2 presents values of E/E' in both groups before and during exercise. On exercise we also observed a late diastolic mitral annular velocity (A') increase only in control group with a significant difference in ΔA' between these two groups (*p* = 0.04). The detailed echocardiographic data is presented in Table 3.

### Reproducibility

All the echocardiographic images were analysed by the same investigator. The intraobserver variability by ICC at rest varied from 0.87 to 0.98 and on exercise from 0.75 to 0.97.

### Multivariate logistic regression analysis

Variables significant in univariate comparisons (p < 0.10) were included into multivariate logistic regression model with bootstrap correction to identify the set of the statistically significant risk factors for dyspnea. Sex and age were added to the model as confounders.

In multivariate analysis PIIINP (unadjusted odds ratio [OR] = 8.2, 95% confidence interval [Cl] 1.7–40.6; *p* = 0.001; adjusted OR = 8.7; 95%CI: 1.5–48.3; p = 0.001.) and E/E' ratio on exercise (unadjusted OR = 1.8, 95%CI: 0.8–4.0; *p* = 0.033; adjusted OR = 2.0; 95%CI: 0.8–4.8; p = 0.012) were the only factors significantly associated with the presence of dyspnea (Table 4).

## Discussion

### Significance of the study

Currently, patients with hypertension are defined as HF group A^3^ despite the absence of signs and symptoms, due to the high risk of developing of the disease. Patients with hypertension, even well controlled, without LVH and with normal resting echocardiography, may have symptoms of HF only during exercise, usually in the form of exertional dyspnea^3^. The origin of the symptoms of myocardial dysfunction in these groups of patients is still unclear. One of the hypotheses involves myocardial stiffness and impaired relaxation^3^.

Various markers of LV systolic and diastolic function derived from Doppler echocardiography have been used to predict LV functional capacity^19^,^20^. However, mitral inflow is dependent on LV relaxation and LA pressure, thus the confounding effects of changes in loading conditions can significantly affect the measurements determined by Doppler recordings of LV filling velocities^19^,^20^. Tissue Doppler imaging (TDI) records systolic and diastolic velocities within the myocardium, and has been shown to provide accurate quantification of regional and global LV function^19^,^20^.

Studies have shown that HFpEF is characterized by an increase in cardiomyocyte stiffness, but also by production and deposition of extracellular matrix^21^,^22^. Cardiac fibrosis is difficult to quantify and in this regard more sensitive biomarkers that are easy to measure are very important^21^,^22^. Therefore we added TDI derived parameters to other standard Doppler echocardiographic measurements and evaluated the levels of selected biochemical biomarkers to find potential predictors of impaired exercise capacity in patients with hypertension.

### Brief description of results

In our study significantly higher concentrations of PIIINP were observed in patients with hypertension with exertional symptoms and in patients with advanced HF compared to the control group, which might suggest that PIIINP may be the first, early biomarker of HF in patients with hypertension. It was the only biomarker that essentially differed between control, dyspnea and HF group. Other investigated biomarkers showed significant differences only between hypertensive patients with normal resting echocardiography (control and dyspnea groups) and patients with advanced HF. Hypertensive patients with preserved EF (control and dyspnea groups) had comparable echocardiographic findings at rest except isovolumic relaxation time (IVRT), which was significantly longer in the dyspnea group (*p* = 0.03). In exertional echocardiography the patients in dyspnea group had lower peak early diastolic mitral annular velocity (E') and peak systolic mitral annular velocity (S') according to TDI and E/E' ratio was significant higher in the symptomatic group as compared to controls. On exercise we also observed the increase of a late diastolic mitral annular velocity (A') in patients without symptoms. Based on multivariate regression analysis, the only independent factors of dyspnea were PIIINP and E/E' ratio on exercise.

### PIIINP as an early biomarker of HF in hypertensive patients

The circulating markers of collagen metabolism such as PIIINP and C-terminal telopeptide of collagen I (CITP) are increased in HFpEF patients while markers of extracellular matrix turnover and matrix degradation such as matrix metalloproteinase 2 (MMP-2) and MMP-9 are reduced^23^. In contrast to HF with reduced EF myocardial stiffness may even be more important than extracellular fibrosis as a mechanism for diastolic stiffness in HFpEF. It has been observed that HFpEF patients with only mild elevations of collagen volume fraction may have highly elevated LV end-diastolic pressures^23^.

PIIINP and collagen I N-terminal propeptide [PINP] must undergo a series of sequential postsynthetic processing steps in order to become a mature structural collagen fibril^23^,^24^. One of these steps is cleavage of PINP and PIIINP. Both the propeptides and telopeptides are of small molecular weight and thereby move from the interstitial space into the vascular compartment, which in turn allows for quantification in a systemic blood sample. In the study of Martos *et al.*^24^ PIIINP was increased in patients with LVH and HF^24^. Cortes *et al.*^25^ reported that serum PINP and PIIINP were increased in LVH compared to non-hypertrophy (*p* = 0.01 and *p* = 0.005, respectively). Moreover, serum PIIINP was associated with sFas (r = 0.386, *p* < 0.0001) and sTNFR1 (r = 0.298, *p* < 0.001)^25^. Similar results were obtained by Agriniera *et al.,* where the authors showed that PIIINP level was higher in the presence of LVH than without LVH (3.2 ± 1.0 *vs.* 2.6 ± 0.8 ng/ml, *p* = 0.001)^26^. In other studies increased PIIINP serum level was found to be associated with LV diastolic dysfunction, LVH and NYHA functional class in hypertensive patients^27^,^28^. In the study of Lopez *et al*., plasma CT-1 and NT-proBNP and selected serum biomarkers of myocardial fibrosis were significantly increased in hypertensive patients with HF^29^. Barasch *et al.*^30^ performed a nested case-control study within the Cardiovascular Health Study and assessed the selected biomarkers at 5- or 9-year follow-up in the group of 880 subjects. In the total study population, elevated PIIINP was associated with incident HFpEF during follow-up (OR per tertile 2.2, 95% CI 1.7–2.8)^30^. In community-based studies, it has been reported that PIIINP levels increased with age and BMI. There were no difference in our study according BMI and age between analysed groups^31^.

All these studies are in accordance with our results and suggest that PIIINP as a useful marker of HF. Our study also revealed that PIIINP is the first early biomarker of the beginning of HFpEF in patients with hypertension and normal resting echocardiography.

### Echocardiographic findings

The genesis of symptoms in patients with HFpEF is unclear. Most investigations of HFpEF have focused on cardiac function at rest although most of these patients are breathless only on exercise^30^. The abnormalities of ventricular function in HFpEF may not be apparent at rest but are more obvious on exercise. With the increase in filling pressure early mitral diastolic inflow velocity (E wave) increases with reduced early diastolic annular velocity (wave E'). The ratio of early mitral diastolic inflow velocity to early diastolic mitral annular velocity (E/E') correlates with the wedge pressure in the pulmonary artery^30^. The TDI of early mitral annulus velocity has been reported to be a relatively preload-independent index of LV relaxation, and the ratio of peak early diastolic mitral inflow velocity to myocardial velocity can be used to estimate LV filling pressure^32^. The longitudinal expansion of the LV is reduced and delayed by slower relaxation and not substantially influenced by LA pressure in the presence of diastolic dysfunction. Thus, in contrast to E, E' provides a consistent measurement of diastolic dysfunction despite increases in LA pressure. The peak rate of LV diastolic filling E is decreased with mild diastolic dysfunction but progressively increased in response to elevation in LA pressure. However, the peak E' wave progressively declines with HF worsening, despite increases in LA pressure and LA to LV pressure gradient^32^. Thus E' (but not E) accurately reflects the progressive slowing of LV relaxation as HF develops^32^. In the study of Ha *et al.*^33^, the increase in E′ with exercise was significantly smaller in patients with hypertrophic cardiomyopathy (*p* = 0.0002). Kitzman *et al.*^34^ observed an increase in LV filling pressure with exercise in patients with HF and preserved systolic function. Similar changes have been reported in normotensive patients with normal LVEF without inducible myocardial ischemia and exaggerated systolic BP response to exercise^35^. However, these studies did not assess the effect of stress on the diastolic indices specifically in patients with HFpEF. In a study by Chattopadhyay *et al.*^36^, 41 patients with hypertension and HF symptoms only on exercise and 29 controls underwent dobutamine stress echocardiography with color TDI. At rest, early diastole annular tissue velocity (E') in the group with exertional dyspnea was similar to controls. The E' decreased and the E/E' ratio increased with stress in the HFpEF but not in controls. The 6-minute walk distance was shorter and negatively correlated to the E/E' ratio at rest and stress in the HFpEF group^36^. The goal of the study by Terzi *et al.*^37^ was to examine whether TDI-derived parameters add incremental value to other standard Doppler echocardiographic measurements in predicting exercise capacity. The study enrolled 59 consecutive patients with stable congestive HF. Systolic and early diastolic velocities of mitral annulus were decreased and the E/E' ratio was increased in the restrictive group as compared to controls, but there was no significant difference in late diastolic velocity and E'/A' ratio between the restrictive group and controls^37^. Interestingly, the patients with the nonrestrictive pattern and E/E' ratio >7.5 had a reduced exercise capacity, as did the group with restrictive LV filling patterns. Univariate analysis demonstrated that the peak S' (r = 0.30, *p* = 0.03), peak E' (r = 0.38, *p* = 0.004) and peak A' (r = 0.35, *p* = 0.009) correlated significantly with maximum exercise capacity^37^. According to multivariate analysis, the index of the E/E′ ratio derived from dividing peak early diastolic mitral inflow velocity by the mitral annular velocity was found to be the most powerful predictor of peak oxygen uptake and the E/E' ratio >7.5 enables the functional limitation to be predicted regardless of the mitral flow profile^37^. The causal relation between exercise intolerance and induced diastolic impairment has also been demonstrated in patients with exercise intolerance and normal LV systolic function^38^. Tan *et al.* assessed patients with symptomatic HF and normal echocardiographic image and the control group at rest and during submaximal exercise standard tissue Doppler and speckle tracking echocardiography^39^. At rest, systolic longitudinal and radial strain, systolic mitral annular velocities, and apical rotation were lower in symptomatic patients, and failed to rise normally on exercise. E′ and E/E′ are influenced by age in healthy subjects. Age-related increases in E/E′ do not seem to be a consequence of higher LV diastolic pressures since left atrial pressure does not significantly rise in healthy elderly subjects. The high values for E/E′ are common in this setting. There were no significant age differences between our groups^20^.

We showed that in exertional echocardiography the patients from the dyspnea group had lower peak early diastolic mitral annular velocity (E'), and E/E' ratio was increased as compared to controls. Our observations are consistent with other presented findings^36^,^37^,^38^,^39^,^40^.

The impaired diastolic reserve results in stress-induced increase in the LV end-diastolic pressure, giving rise to exercise intolerance^36^,^37^,^38^,^39^,^40^,^41^,^42^.

### Study limitations

There are several limitations of this study. We assessed myocardial velocities measured by pulsed TDI and not color-coded TDI. A limitation of this technique is the possibility to measure the velocity of only one area at a given time. The great advantage, however, is its availability even in the older generation ultrasound systems, and that it does not require post-processing work, which makes it more useful in everyday medical practice. The diastolic blood flow and annular velocities were measured at submaximal heart rates to avoid the problem of fusion of these velocities at peak stress. Our echocardiographic results were not compared with results of cardiopulmonary exercise testing. The study involved also a relatively small number of patients and the findings need to be confirmed in a larger population.

### Conclusions

In conclusion, PIIINP is the first early biomarker for the development of HF in patients with hypertension and normal resting echocardiography. Patients with treated hypertension with normal resting echocardiography can have exercise intolerance or breathlessness and worse LV relaxation on exercise. Exertional echocardiography using TDI may indicate patients with incipient HFpEF and increase the diagnostic value of echocardiography in HFpEF diagnosis.

The issue of exertional dyspnea and HF with normal ejection fraction in patients with hypertension and normal results of resting echocardiography is a new problem in the world. So far, there are no treatment recommendations for this group of patients. An important issue is the identification of patients with hypertension at risk of developing this syndrome, the evaluation of new biochemical markers and new methods of diagnosis in this group of patients. There are only few studies available on this topic separately analyzing echocardiography issues and usefulness of single biomarkers, thus our project seems to be very novel and pioneering. We revealed that circulating biomarkers of extracellular matrix remodelling and exercise echocardiography could be an inexpensive and simple diagnostic panel for the prediction of HF in patients with hypertension.

## Figures and Tables

**Table 1:** Characteristics of groups of patients without symptoms (control), with exertional dyspnea (dyspnea) and with clinical heart failure

	SD			*p*		
Parameter	Control Group N = 22	Dyspnea Group N = 27	Heart Failure Group N = 50	Control vs Dyspnea	Control vs Heart Failure	Dyspnea *vs* Heart Failure
**Age (years)**	59.5 ± 13	63.5 ± 9	64.5 ± 11	ns	ns	ns
**Duration of hypertension (years)**	12.4 ± 7	9.3 ± 6	13.9 ± 6	ns	ns	ns
**BMI kg/m^2^**	27.0 ± 5	27.6 ± 4	28.6 ± 4	ns	ns	ns
**GFR MDRD (ml/min/1,73 m^2^)**	88.4 ± 8	90 ± 6	67.7 ± 24	ns	0.0009	0.0001
**Systolic BP mmHg**	136 ± 7	135 ± 9	122 ± 15	ns	0.0001	0.0001
**Diastolic BP mmHg**	79 ± 9	83 ± 7	75 ± 8	ns	ns	0.001
**HR**	71 ± 6	70 ± 6	74 ± 9.8	ns	ns	ns
**Hemoglobin**	14.3 ± 0.9	14.4 ± 1	13.8 ± 1	ns	ns	ns
**Hematocrit**	41.4 ± 3	41.4 ± 4	42.5 ± 4	ns	ns	ns
**Na mmol/l**	140.9 ± 4	138.8 ± 3	138.4 ± 3	ns	ns	ns
**K mmol/l**	4.3 ± 0.5	4.1 ± 0.4	4.3 ± 0.4	ns	ns	ns
**Creatinine**	0.8 ± 0.24	0.9 ± 0.28	1.1 ± 0.29	ns	ns	ns
**CRP mg/l**	2.3 ± 1	2.2 ± 1.4	3.6 ± 4.7	ns	ns	ns
**TNF-alpha (pg/ml)**	28.45 ± 23	36.46 ± 57	30.94 ± 16	ns	ns	ns
**IL1R1(ng/ml)**	0.37 ± 0.2	0.52 ± 0.3	0.35 ± 0.1	ns	ns	ns
**TGF-beta 1(ng/ml)**	11.55 ± 2	9.96 ± 3	5.98 ± 2.5	ns	<0.0001	<0.0001
**Syndecan (ng/ml)**	1.19 ± 1	1.54 ± 1	4.14 ± 3.3	ns	<0.0001	0.0015
**Galectin-3 (ng/ml)**	22.27 ± 6	20.45 ± 4	18.59 ± 11	ns	ns	ns
**Cardiotrophin (pg/ml)**	77.66 ± 81	98.04 ± 136	229.51 ± 129	ns	<0.0001	<0.0001
**NT-proBNP (pg/ml)**	146.68 ± 102	152.93 ± 126	1889 ± 3368	ns	<0.0001	<0.0001
**Cystatin C (mg/l)**	0.77 ± 0.3	0.84 ± 0.5	1.37 ± 0.8	ns	0.0004	0.0001
**Lipocalin-2 NGAL (ng/ml)**	63.00 ± 37	66.56 ± 35	50.71 ± 45	ns	ns	0.0360
**PIIINP (ng/ml) Median**	1.27 ± 1.03	2.9 ± 0.94	2.62 ± 0.97	0.0001	0.0001	ns
**(Q25–Q75)**	1.3 (0.3–2.0)	2.9 (2.2–3.6)	2.6 (1.9–3.3)			
				Chi-square		*P*
**Gender (male)**	9(41)	13(48)	43(86)	19.7		0.0001
**Smoking**	2(9)	2(7)	2(4)	0.81		ns
**Heart Failure acc. NYHA**				73.0		0.0001
**I**	0	14(51)	5(10)			
**II**	0	13(48)	21(42)			
**III**	0	0	24(48)			
**IV**	0	0	0			
**Stenocardia acc. CCS**				85.5		0.0001
**0**	21(95)	6(22)	2(4)			
**I**	1(5)	4(15)	34(68)			
**II**	0	17(63)	13(26)			
**III**	0	0	1(2)			
**Diabetes mellitus or abnormal glucose level**	2(9)	5(18)	8(16)	0.96		ns
**Statins**	69(27)	15(55)	32(64)	8.5		0.0141
**Insulin**	2(10)	2(7)	3(6)	0.2		ns
**Diuretics**	8(36)	13(48)	46(92)	30.4		0.0001
**Beta -blockers**	16(72)	22(81)	48(96)	8.5		0.013
**Spironolactone/eplerenone**	2(9)	5(18)	41(82)	50.7		0.0001
**Acetylsalicylic acid**	4(18)	13(49)	26(53)	8.3		0.015
**ACE-inhibitors**	11(50)	11(40)	43(86)	19.8		0.0001
**ARBs**	10(45)	12(44)	8(16)	10.0		0.006
**CCB**	10(45)	6(22)	4(8)	12.8		0.001
**Digoxin**	0	0	12(24)	18.0		0.0001

**ABBREVIATIONS:** BMI, body mass index; GRF MDRD, glomerular filtration rate on the basic of the study Modification of Diet in Renal Disease; BP, blood pressure; HR, heart rate; Na, sodium; K, potassium; NYHA, New York Heart Association classification for heart failure; CCS, Canadian Cardiovascular Society; TGF-beta 1, transforming growth factor beta 1; NT-proBNP, N-terminal pro-brain natriuretic peptide; NGAL, neutrophil gelatinase-associated lipocalin; PIIINP, collagen III N-terminal propeptide; TNF alpha, tumor necrosis factor alpha; IL1R1, interleukin 1 receptor, type I, ACE-inhibitors, angiotensin-converting enzyme inhibitors; ARB, angiotensin receptor blocker; CCB – calcium channel blocker.

**Table 2:** Resting echocardiography parameters in each group

	Mean ± standard deviation (SD)			*p*		
Parameter	Control Group (n = 22)	Dyspnea Group (n = 27)	HF Group (n = 50)	Control *vs* Dyspnea	Control *vs* Heart Failure	Dyspnea *vs* Heart Failure
**LVEDD (mm)**	50.8 ± 6	49.0 ± 5	63.2 ± 9.5	ns	<0.0001	<0.0001
**LVESD (mm)**	30.5 ± 5	32.5 ± 6	48.1 ± 10	ns	<0.0001	<0.0001
**LVEF (%)**	62.5 ± 4	59.5 ± 3	36.7 ± 10	ns	<0.0001	<0.0001
**LA (mm)**	35.9 ± 5	37.1 ± 4	45.1 ± 7.7	ns	<0.0001	<0.0001
**Dilated LA (%)**	36	26	74	ns	<0.001	<0.001
**peak E cm/s**	70.1 ± 11	71.3 ± 18	62.9 ± 23	ns	ns	ns
**peak A cm/s**	65.3 ± 23	70.3 ± 16	87.4 ± 13	ns	0.019	ns
**E/A ratio**	1.1 ± 0.4	1.0 ± 0.3	0.6 ± 0.2	ns	0.012	ns
**DT (ms)**	246.2 ± 58	267.7 ± 72	343.1 ± 106	ns	ns	Ns
**LV mass (g)**	193.0 ± 51	194 ± 61	460 ± 244	ns	0.001	0.001
**LVMI**	104.2 ± 31	103.7 ± 24	240 ± 132	ns	0.001	0.001
**IVSD (mm)**	9.5 ± 1	9.3 ± 1	11.7 ± 2.3	ns	0.003	<0.0001
**PWD (mm)**	9.1 ± 1	9.3 ± 1	11.3 ± 2.8	ns	0.019	0.019
**RVdD (mm)**	27.9 ± 3	26.8 ± 3	28.8 ± 4.8	ns	ns	ns
**LVEDV (ml)**	85.8 ± 25	80.6 ± 21	213.5 ± 60	ns	<0.0001	<0.0001
**LVESV (ml)**	29.8 ± 7	28.2 ± 8	135.5 ± 50	ns	<0.0001	<0.0001
**TAPSE (mm)**	25.9 ± 3	24.5 ± 3	21.6 ± 3.6	ns	0.004	ns
**IVRT (ms) Rest**	89.4 ± 26	104.8 ± 19	105.1 ± 15	0.03	0.03	ns
**E' Rest (cm/s)**	10.5 ± 2	10.7 ± 2	6.4 ± 3	ns	0.01	0.01
**A' Rest (cm/s)**	10.8 ± 3	11.3 ± 3	11.8 ± 2	ns	ns	ns
**E/E' ratio Rest**	7.0 ± 2	7.0 ± 1	10.6 ± 5	ns	0.02	0.02
**S' rest (cm/s)**	9.0 ± 2	9.0 ± 2	6.0 ± 3	ns	0.02	0.02

**ABBREVIATIONS:** LVEDD, left ventricular end-diastolic diameter; LVESD, left ventricular end-systolic diameter; LVEF, left ventricular ejection fraction; LA, left atrial diameter; E, early mitral diastolic inflow velocity; A, late mitral diastolic inflow velocity; E/A, ratio of early to late mitral inflow velocities; DT, deceleration time of peak early mitral filling velocity; LVMI, left ventricular mass index; IVSD, diastolic interventricular septal thickness; PWD, diastolic posterior wall thickness; RVDD, right ventricular diastolic diameter; LVEDV, left ventricular end-diastolic volume; LVSV, left ventricular systolic volume; TAPSE, tricuspid annular plane systolic excursion; IVRT, isovolumic relaxation time; E', early diastolic annular velocity; A', late diastolic annular velocity; E/E', ratio of early mitral diastolic inflow velocity to early diastolic mitral annular velocity; S', systolic mitral annular velocity

**Table 3:** Evaluation of selected parameters in control group and in dyspnea group during exercise

	Mean ± standard deviation (SD)		p
Parameter	Control Group (n = 22)	Dyspnea Group (n = 27)	Control *vs* Dyspnea
**Efficiency in MET**	8.2 ± 1	7.1 ± 1	ns
**Systolic BP mmHg Ex**	160 ± 18	165 ± 19	ns
**Diastolic BP mmHg Ex**	85 ± 10	87 ± 12	ns
**HR Ex**	91 ± 5	93 ± 6	ns
**peak E Ex (cm/s)**	85.5 ± 21	88.7 ± 19	ns
**peak A Ex (cm/s)**	74.9 ± 25	73.5 ± 18	ns
**E/A ratio Ex**	1.2 ± 0.5	1.2 ± 0.4	ns
**DT Ex (ms)**	239.3 ± 78	260 ± 70	ns
**E' Ex (cm/s)**	14.9 ± 3	12.8 ± 1	0.020
**Δ E'**	4.4 ± 3.76	2.2 ± 2.41	0.004
**A' Ex (cm/s)**	12.0 ± 4	10.7 ± 3	ns
**Δ A'**	1.2 ± 3.41	−0.6 ± 2.46	0.040
**E/E' ratio Ex**	5.1 ± 1	6.7 ± 1	0.034
**S' Ex (cm/s)**	10.9 ± 2	9.3 ± 2	0.017

**ABBREVIATIONS:** MET, metabolic equivalent defined as the amount of oxygen consumed while sitting at rest and is equal to 3.5 ml O_2_ per kg body weight x min; Ex, on exercise; BP, blood pressure; HR, heart rate E', early diastolic annular velocity; Δ E' = E' Ex - E' rest; A', late diastolic annular velocity; Δ A' = A' Ex - A' rest; E/E', ratio of early mitral diastolic inflow velocity to early diastolic mitral annular velocity; S', systolic mitral annular velocity; Ex, on exercise.

**Table 4:** Bootstrapped logistic regression results

	unadjusted				adjusted for sex and age			
Parameter	OR	95%CI for OR		*p - value*	OR	95%CI for OR		*p - value*
**IVRT (ms) Rest**	1,039	0,970	1,112	0,149	1,047	0,965	1,136	0,066
**E' Ex (cm/s)**	1,103	0,578	2,105	0,403	1,256	0,552	2,859	0,141
**E/E' ratio Ex**	1,803	0,814	3,994	0,033	2,016	0,844	4,816	0,012
**S' Ex (cm/s)**	0,901	0,470	1,725	0,394	0,773	0,365	1,638	0,106
**Δ E'**	0,920	0,546	1,551	0,394	0,856	0,450	1,628	0,176
**Δ A'**	0,879	0,487	1,585	0,379	0,862	0,454	1,638	0,161
**PIIINP**	8,203	1,657	40,618	0,001	8,653	1,549	48,319	0,001
**Model description**	Model is statistically significant: χ^2^ = 32.72; df = 7; p<0.001; Nagelkerke's R^2^ = 0.72; Hosmer – Lemeshaw test: χ^2^ = 5.57; df = 8; p = 0.696				Model is statistically significant: χ^2^ = 33.41; df = 9; p < 0.001; Nagelkerke's R^2^ = 0.73; Hosmer – Lemeshaw test: χ^2^ = 8.11; df = 8; p = 0.423			
